# Physicochemical Characterization, Antimicrobial and Antibiofilm Activities of *Thymus vulgaris* and *Rosmarinus officinalis* Essential Oil Nanoemulsions with Potential Mouthwash Applications

**DOI:** 10.3390/ph19081229

**Published:** 2026-08-04

**Authors:** Cemre Irem Ayguler, Aleyna Ozveren, Timur Hakan Barak, Gamze Benli Yardimci, Ipek Tekin, Mujde Eryilmaz

**Affiliations:** 1 Department of Pharmaceutical Technology, Faculty of Pharmacy, Acibadem Mehmet Ali Aydinlar University, Istanbul 34752, Türkiye; cemre.ayguler@acibadem.edu.tr; 2 Department of Pharmaceutical Microbiology, Faculty of Pharmacy, Acibadem Mehmet Ali University, Istanbul 34752, Türkiye; aleyna.ozveren@acibadem.edu.tr (A.O.); ipek.tekin@live.acibadem.edu.tr (I.T.); 3 Department of Pharmacognosy, Faculty of Pharmacy, Acibadem Mehmet Ali Aydinlar University, Istanbul 34752, Türkiye; timur.barak@acibadem.edu.tr; 4 Department of Pharmaceutical Microbiology, Faculty of Pharmacy, Afyonkarahisar Health Sciences University, Afyonkarahisar 03030, Türkiye; gamze.benli@afsu.edu.tr

**Keywords:** essential oil nanoemulsion, mouthwash formulation, oral biofilm, *Streptococcus mutans*, *Thymus vulgaris*, *Rosmarinus officinalis*

## Abstract

**Background/Objectives**: In this study, nanoemulsions containing *Thymus vulgaris* (TV) and *Rosmarinus officinalis* (RO) essential oils (EOs) were prepared and characterized, and their antibacterial, antifungal, and antibiofilm activities were evaluated against some oral pathogens. **Methods**: Three nanoemulsions containing 5% TV-EO (F1), 5% RO-EO (F2), and a combination of 2.5% TV-EO and 2.5% RO-EO (F3) were prepared by emulsification followed by high-speed homogenization. **Results**: Gas chromatography–mass spectrometry coupled with flame ionization detection (GC-MS-FID) analysis revealed linalool (79.51%) as the major constituent of TV-EO and eucalyptol (58.13%), together with camphor (10.80%), as the predominant compounds of RO-EO. All nanoemulsions exhibited nanosized droplets, low polydispersity index (PDI) values (<0.3), and moderate to high colloidal stability. Transmission electron microscopy (TEM) analysis revealed predominantly spherical droplets in all formulations (F1–F3). All formulations exhibited antimicrobial activity against *Streptococcus mutans*, *Lactobacillus acidophilus*, *Enterococcus faecalis*, and *Candida albicans*. Among the tested formulations, F2 showed the strongest antibacterial activity, whereas F3 exhibited the highest antibiofilm activity against mature *S. mutans* biofilms, achieving approximately 80% inhibition, comparable to chlorhexidine (CHX). Although F3 possessed the smallest droplet size, its antimicrobial activity was lower than that of F2, suggesting that biological activity was influenced not only by droplet size but also by EO composition. **Conclusions**: These findings demonstrate the potential of TV-EO and RO-EO nanoemulsions as promising natural alternatives for mouthwash formulations and highlight the importance of EO composition in determining antimicrobial and antibiofilm efficacy.

## 1. Introduction

The oral cavity provides a warm, humid, and nutrient-rich environment that supports microbial colonization. Microbial colonization results from the complex and dynamic interaction among microorganisms, the host, and dietary variables, which in turn triggers the formation of pathogenic biofilms. Oral biofilms, which develop on tooth surfaces and dental materials, are recognized as major virulence factors involved in the pathogenesis of dental caries, periodontitis, and endodontic infections. Due to their structural complexity and protective extracellular matrix, biofilms exhibit increased resistance to antimicrobial agents and host defense mechanisms, posing a major challenge for oral disease management [1,2,3].

Chlorhexidine (CHX) remains the gold standard for plaque control. However, its long-term use is associated with several adverse effects that limit patient compliance, including tooth discoloration, taste disturbances, mucosal irritation, and tartar formation. These drawbacks have increased interest in natural compounds as safer alternatives for oral health care [4,5].

Essential oils (EOs), which give plants their distinct flavor and aroma, are typically complex mixtures of volatile organic compounds that are biosynthesized as secondary metabolites. Since ancient times, many cultures have utilized EOs for their various biological activities, such as antimicrobial, antifungal, anti-inflammatory, and antioxidant properties [6,7]. However, several issues, such as their high volatility and high potential for decomposition upon direct exposure to heat, humidity, light, or oxygen, restrict their application [8].

Among EOs, *Thymus vulgaris* (TV) and *Rosmarinus officinalis* (RO) have attracted considerable attention due to their rich phytochemical compositions and broad-spectrum biological activities. TV-EO has been widely reported to possess antibacterial, antifungal, antioxidant, and anti-inflammatory properties, and its antimicrobial effects are commonly associated with phenolic and monoterpenoid constituents such as thymol, carvacrol, γ-terpinene, p-cymene, and linalool [9,10,11,12]. Previous studies have demonstrated the inhibitory activity of TV-EO against clinically relevant oral pathogens, including *Streptococcus mutans* and *Candida albicans*, supporting its potential use in oral care formulations [13,14]. RO-EO is also known for its antimicrobial, antioxidant, and anti-inflammatory properties, mainly attributed to monoterpenes such as 1,8-cineole, camphor, α-pinene, borneol, and related compounds [15,16,17]. Antimicrobial activity of RO-derived preparations has been reported against oral pathogens such as *S. mutans* and *Enterococcus faecalis*, while RO-EO has also shown antifungal activity against *C. albicans* [18,19]. The antimicrobial activity of EO terpenoids is largely attributed to their ability to interact with microbial lipid membranes, increasing membrane permeability and disrupting membrane integrity, ultimately leading to leakage of intracellular constituents and cell death [20]. Currently, the mouthwash Listerine^®^ is widely used. In the beginning, Listerine^®^ was composed of four EOs: wintergreen, thyme, eucalyptus, and peppermint. Then, the Listerine^®^ formula was altered. Menthol, eucalyptol, thymol, and methyl salicylate—the main components of each EO—were extracted and mixed. They were dissolved in an ethanol–water mixture. These days, synthetic derivatives have replaced methyl salicylate and menthol. EOs are not present in Listerine^®^; instead, it contains individual components [21,22].

Nanoemulsions are a combination of oil and water phases that are stabilized using surfactants and contain droplets smaller than 200 nm [23,24]. Their smaller droplets have a larger surface area, which increases absorption [25]. EO nanoemulsion enables the effective encapsulation and delivery of EOs. EO nanoemulsions have been demonstrated to have antimicrobial activity against a wide range of microorganisms. EOs contain bioactive compounds that have antimicrobial effects because they can damage microorganism cell membranes and inhibit them from growing [26].

In the present study, nanoemulsions containing TV-EO and RO-EO were developed as potential mouthwash formulations. Unlike commercially available mouthwash Listerine^®^, which contains isolated EO constituents rather than whole EOs, the developed nanoemulsions incorporated the complete EOs. This approach represents a novel strategy for potential mouthwash formulation by utilizing whole EOs instead of isolated EO constituents.

Although several studies have investigated the antimicrobial properties of individual EOs, comparative studies evaluating nanoemulsions prepared from TV-EO, RO-EO, and their combination for mouthwash applications remain limited. Furthermore, information on their effects on mature oral biofilms is scarce. Therefore, in this study, nanoemulsions containing EOs from TV and RO were prepared and characterized with respect to droplet size and distribution, zeta potential, morphological analysis, antimicrobial activity, and antibiofilm activity.

## 2. Results

### 2.1. Gas Chromatography–Mass Spectrometry Coupled with Flame Ionization Detection (GC-MS-FID) Analysis of EOs

**Figure 1:**
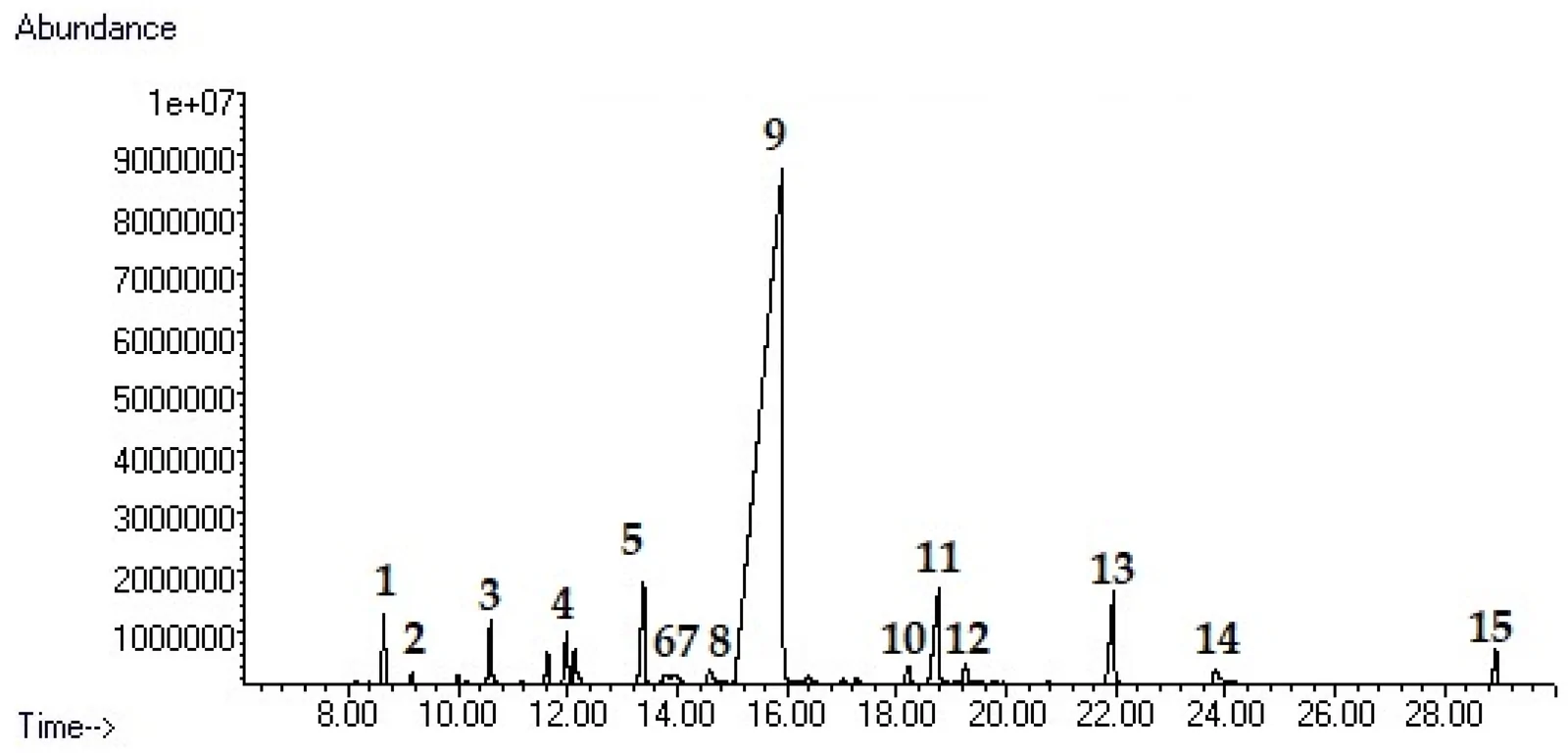
GC-MS-FID chromatogram of the TV-EO.

**Figure 2:**
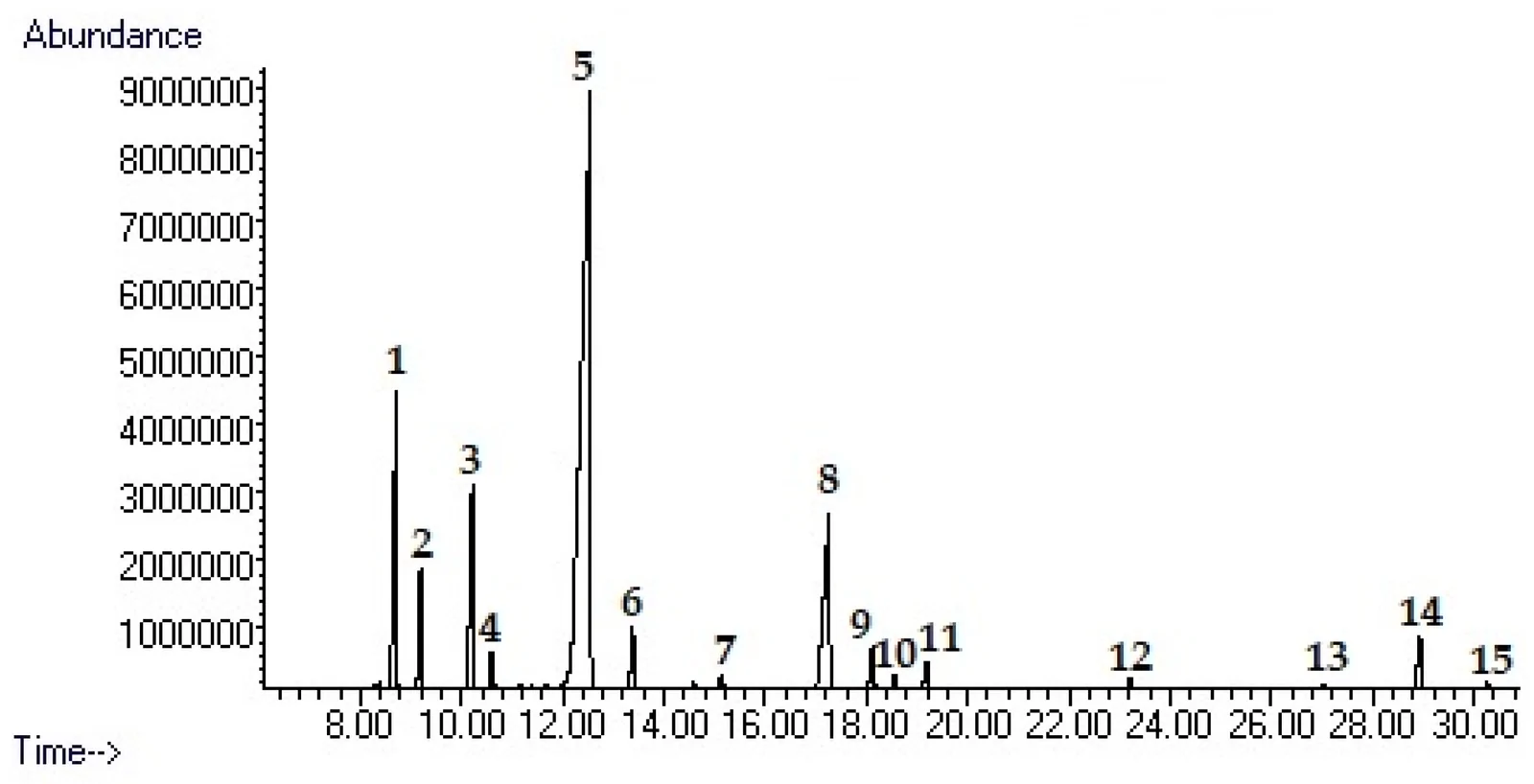
GC-MS-FID chromatogram of the RO-EO.

The results of the phytochemical analysis of TV-EO and RO-EO are given in Table 1 and Table 2, and Figure 1 and Figure 2. Results showed that 97.81% of the phytochemical ingredients of TV-EO were revealed, and 15 different compounds were detected. Linalool was determined as the dominant major ingredient with 79.51 ± 0.98%, which is consistent with its chemotype. γ-Terpinene, terpinene-4-ol, and linalyl acetate were likewise detected in significant amounts.

Phytochemical analysis of RO-EO demonstrated 98.72% of the ingredients and 58.13 ± 0.04% of the EO is eucalyptol. In addition, camphor and α-pinene were detected in significant amounts (10.80 ± 0.03% and 10.09 ± 0.02%, respectively). The results revealed the complex nature of the EOs with compounds from various groups such as monoterpenes and sesquiterpenes. These findings establish a foundational basis for elucidating the potential bioactivities of the samples.

### 2.2. Droplet Size, Polydispersity Index (PDI), and Zeta Potential

Table 3 demonstrates the droplet size, PDI, and zeta potential of nanoemulsions as mean ± standard deviation (SD).

### 2.3. Morphological Characterization of the Nanoemulsions

**Figure 3:**
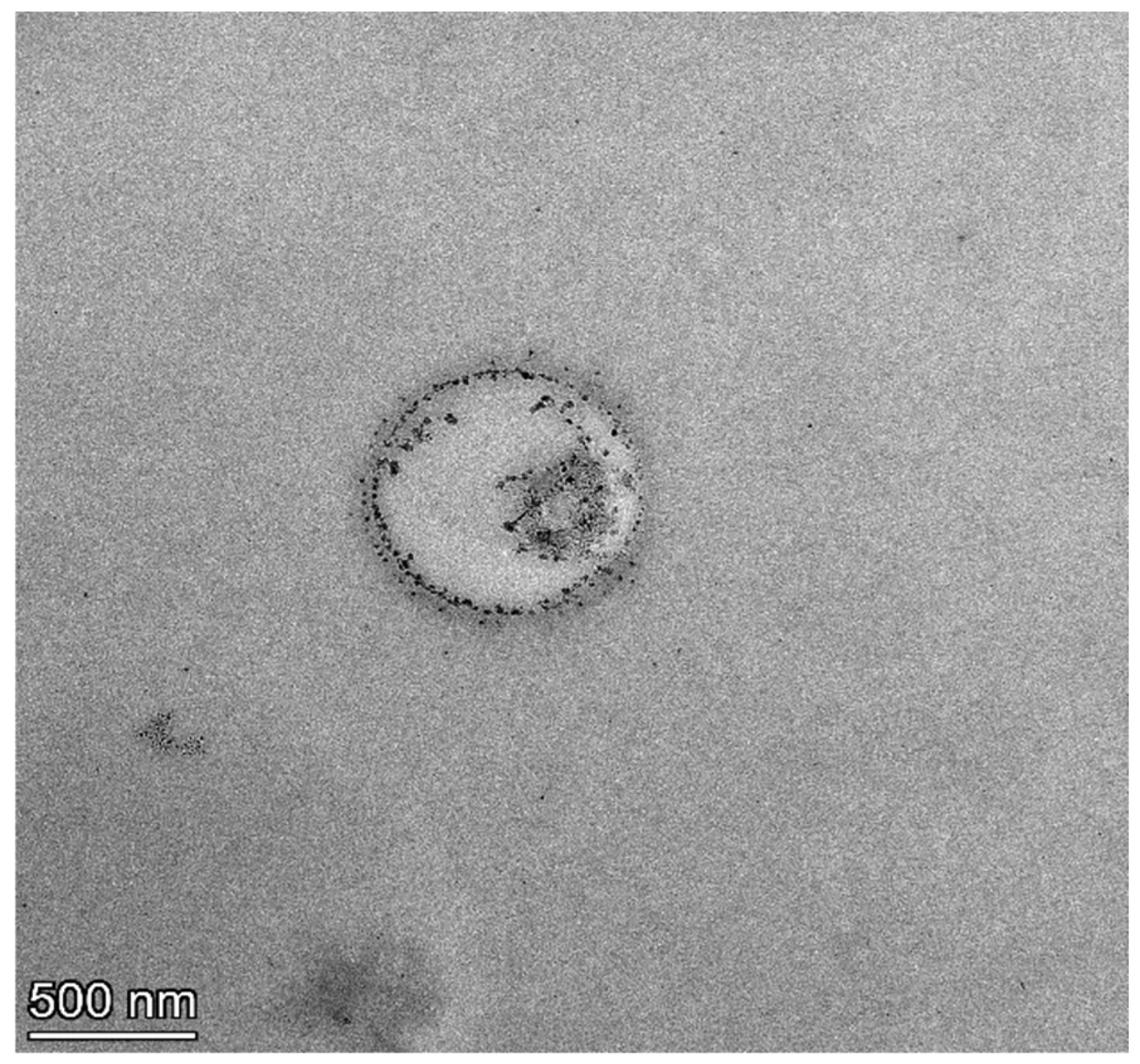
TEM image of F1 (17.3k× magnification).

**Figure 4:**
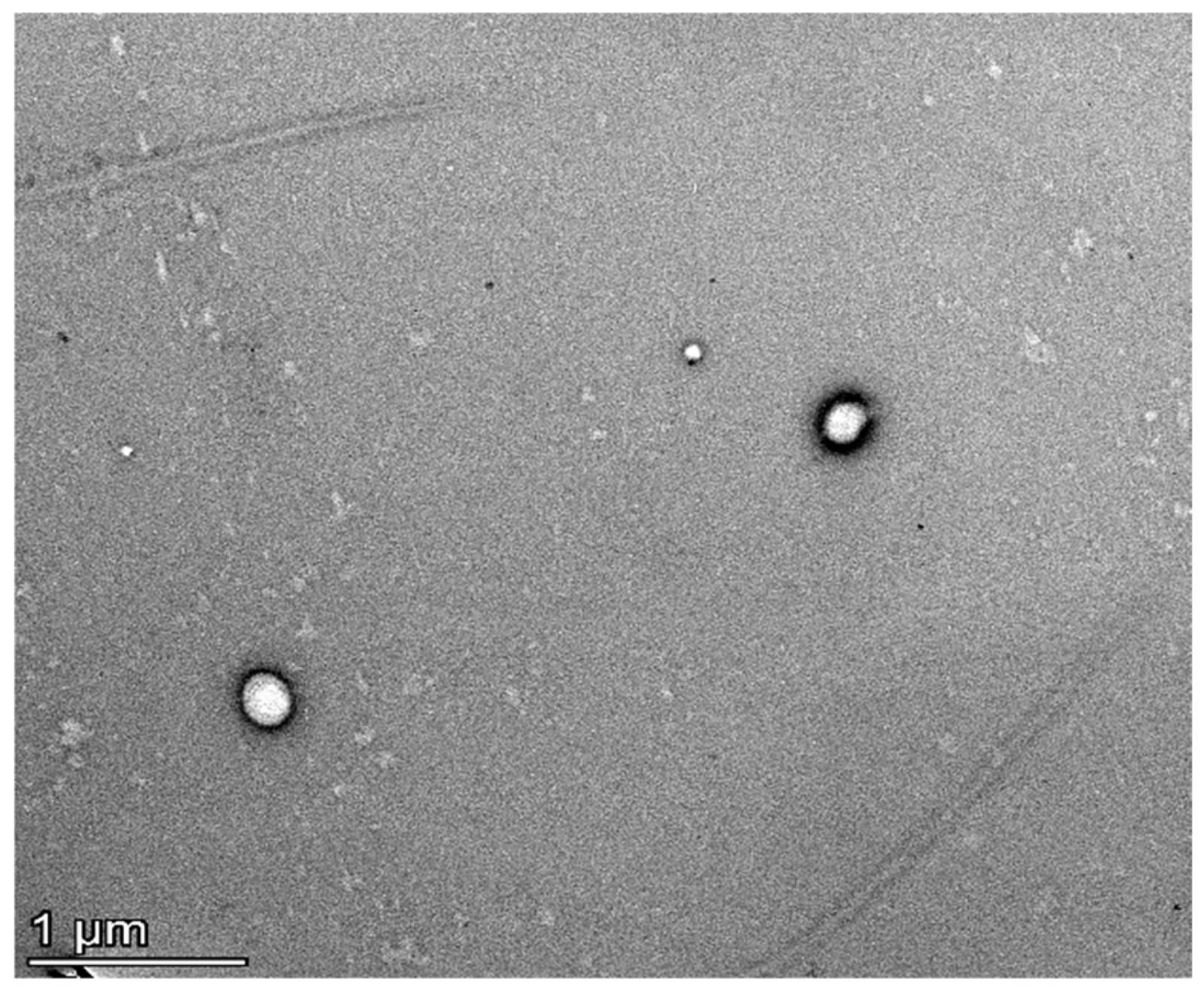
TEM image of F2 (10.9k× magnification).

**Figure 5:**
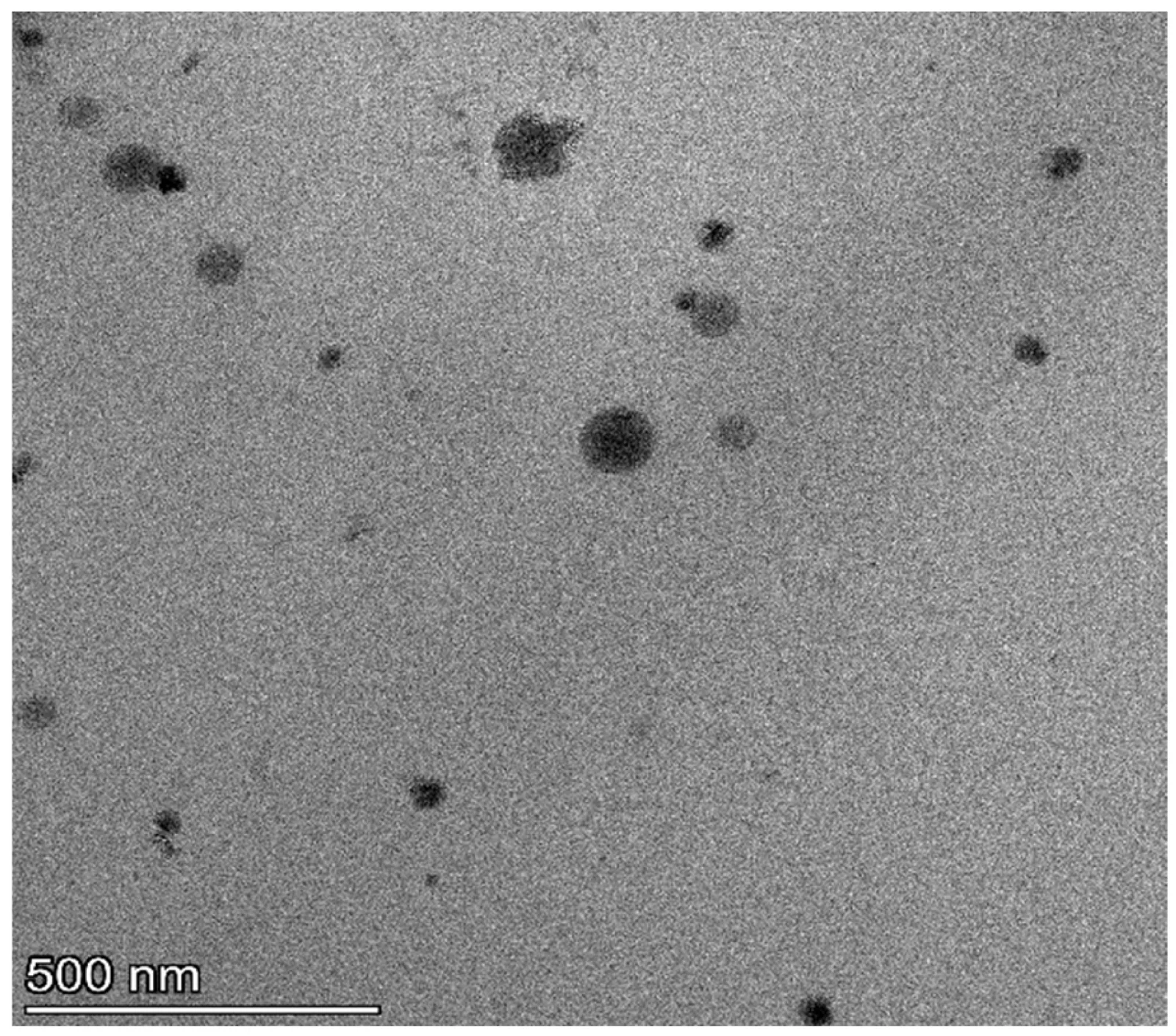
TEM image of F3 (35.5k× magnification).

The transmission electron microscopy (TEM) images of formulations F1, F2, and F3 are presented in Figure 3, Figure 4 and Figure 5.

### 2.4. Antimicrobial Activity of Essential Oils and Nanoemulsions

The minimum inhibitory concentration (MIC) values (mg/mL) of TV-EO, RO-EO, and nanoemulsion formulations are presented in Table 4 and Table 5. The 5% DMSO (dimethyl sulfoxide) negative control did not exhibit antimicrobial activity against any of the tested microorganisms.

### 2.5. Antibiofilm Activity of Essential Oils and Nanoemulsions

**Figure 6:**
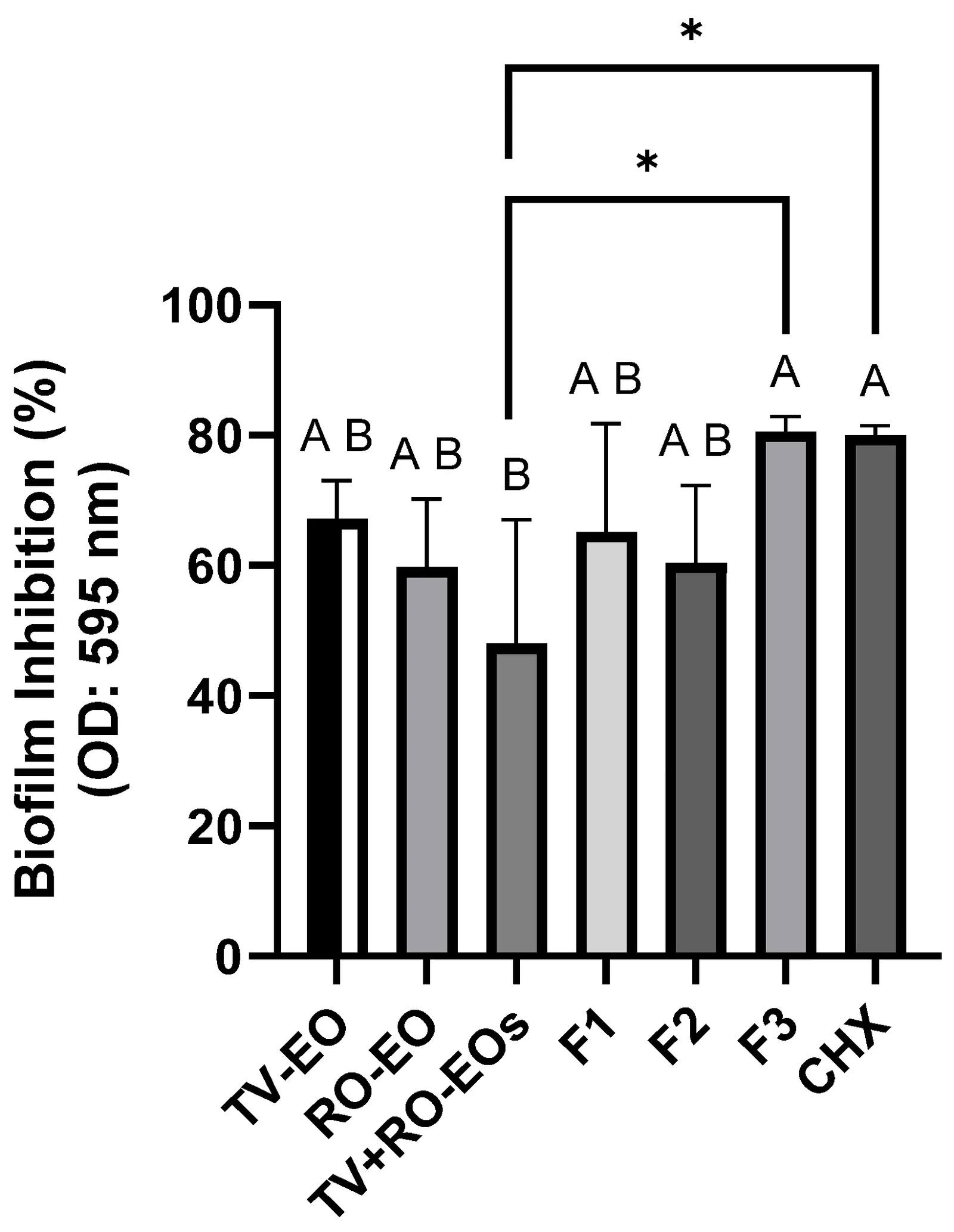
The percentage biofilm inhibition values of TV-EO, RO-EO, and nanoemulsion formulations. Data are expressed as mean ± SD from four independent experiments (n = 4). Statistical differences among groups were evaluated using one-way ANOVA followed by Tukey’s multiple comparison test. Groups sharing the same letter are not significantly different, whereas different letters indicate significant differences (*p* < 0.05). Asterisks indicate statistically significant differences between the connected groups (*p* < 0.05).

The percentage biofilm inhibition values of TV-EO, RO-EO, and nanoemulsion formulations are presented in Figure 6. The corresponding numerical values (mean ± SD) are summarized in Table 6.

Among the tested nanoemulsion formulations, F3 exhibited the highest biofilm inhibition (80.55 ± 2.33%), whereas F2 showed the lowest inhibition (60.35 ± 11.95%). The positive control (CHX) exhibited a biofilm inhibition of 79.95 ± 1.49%. One-way ANOVA followed by Tukey’s multiple comparisons test revealed a significant overall difference among the experimental groups (*p* = 0.0079). However, no statistically significant differences were observed among F1, F2, and F3 or between any of the nanoemulsion formulations (F1–F3) and CHX (*p* > 0.05), whereas the TV + RO-EO combination showed significantly lower biofilm inhibition than both F3 and CHX (*p* < 0.05).

## 3. Discussion

In this study, nanoemulsions prepared using TV-EO, RO-EO, and their combinations were evaluated as potential mouthwash formulations for their physicochemical, antibacterial, antifungal, and antibiofilm activities. All formulations contained the same total EO concentration (5%, *w*/*v*), with identical surfactant concentration and aqueous phase volume. The formulations differed only in EO composition, with F1 containing 5% TV-EO, F2 containing 5% RO-EO, and F3 containing 2.5% TV-EO and 2.5% RO-EO. The formulations exhibited different physicochemical properties and biological activities.

GC-MS-FID analysis revealed marked differences in the phytochemical composition of the investigated EOs. In the present study, GC-MS-FID analysis revealed a linalool-dominant profile for TV-EO, which is consistent with its chemotype, whereas eucalyptol and camphor were the predominant constituents of RO-EO. This compositional profile provides a chemical basis for evaluating these oils, individually and in combination, as potential natural antimicrobial and antibiofilm agents for mouthwash applications. TV-EO was characterized by a linalool-dominant profile, with linalool accounting for 79.51% of the total composition, whereas RO-EO was mainly composed of eucalyptol (58.13%) and camphor (10.80%). Previous studies have demonstrated that the chemical composition of both TV and RO EOs may vary considerably depending on geographical origin, environmental conditions, harvesting period, and extraction methods [27]. In particular, several chemotypes of TV have been described, including thymol-, carvacrol-, geraniol-, and linalool-rich types. Therefore, the biological activities observed in the present study should be interpreted in the context of the identified phytochemical composition rather than solely based on plant species. Our results are consistent with the previous literature. Satyal et al. determined the linalool content of linalool-type TV-EO as 76.15%, while Horvath et al. showed 69.2% and Schmidt et al. reported 68.5% [12,28,29]. Similarly, our results are consistent with the literature for RO-EO. Demirci et al. reported 62.7% eucalyptol, 8.3% camphor, and 12.6% α-pinene, while Bekhechi et al. showed 53.1% eucalyptol, 11.6% camphor, and 7.1% α-pinene [30,31]. Linalool, eucalyptol, and camphor have all been reported to exert antimicrobial effects through interactions with microbial membranes, leading to increased membrane permeability and disruption of cellular integrity. Therefore, the predominance of these compounds suggests that they may have contributed to the antimicrobial and antibiofilm activities observed in this study.

Droplet size and distributions of nanoemulsions were measured, and the results were presented in Table 3. In general, the nanoemulsion droplet size is between 20 and 200 nm [25]. In our study, the droplet sizes of nanoemulsions F1, F2, and F3 were measured as 161.18 ± 3.24 nm, 111.03 ± 0.69 nm, and 55.40 ± 0.44 nm, respectively. These results indicate that the droplet sizes of the nanoemulsions are consistent with the literature. In comparison to F1, F2 produced smaller droplet sizes. When the two EOs were combined (F3), the lowest droplet size was obtained. Although the total EO concentration was kept constant at 5% across all formulations, significant differences in droplet size were observed. This suggests that droplet formation is primarily governed by EO composition rather than total EO concentration. It has been reported that the droplet size of nanoemulsions is strongly influenced by the type of oil phase and its physicochemical properties, particularly interfacial tension and viscosity [32]. Ostertag et al. developed nanoemulsions containing various oil types, including medium-chain triglycerides (MCT), flavor oils (orange and limonene), and long-chain triglycerides such as olive, grape, sesame, peanut, and canola oils. Significant differences in droplet size were observed among the different oil types. They suggested that the viscosity of the oil phase may influence surfactant transport and droplet disruption during emulsification, whereas interfacial tension may affect the mobility of the oil–water interface and droplet breakup [33]. In our study, the combination of two chemically distinct EOs may have altered the physicochemical properties of the oil phase, particularly its interfacial behavior, thereby facilitating droplet disruption during homogenization and contributing to the formation of smaller droplets. This may be attributed to differences in the molecular structure and interfacial behavior of the two EOs.

In basic terms, PDI is a way to represent the size population distribution in a certain sample. A PDI value of 0.3 or lower is generally regarded as acceptable and reflects a homogeneous population [34]. In our study, the PDI values of formulations F1, F2, and F3 were determined as 0.252 ± 0.020, 0.215 ± 0.013, and 0.284 ± 0.009, respectively. Therefore, it can be concluded that the size distribution was homogeneous in all formulations.

Zeta potential values of nanoemulsions were measured, and the results are presented in Table 3. The drug delivery literature frequently classifies dispersions having zeta potential values of ±0–10 mV, ±10–20 mV, ±20–30 mV, and >±30 mV as extremely unstable, relatively stable, moderately stable, and extremely stable, respectively [35]. In our study, the zeta potential values of F1, F2, and F3 were found to be −46.8 ± 2.2 mV, −34.5 ± 5.2 mV, and −21.2 ± 1.6 mV, respectively. The findings revealed that formulations F1 and F2 show high colloidal stability, and formulation F3 exhibits moderate colloidal stability.

In the study conducted by Pavlović et al., the droplet size, PDI, and zeta potential of the nanoemulsion containing 4% RO-EO were reported as 176.8 nm, 0.049, and −2.48 mV, respectively [36]. In contrast, F2 exhibited a smaller droplet size (111.03 nm), a higher PDI (0.215), and a higher absolute zeta potential (−34.5 mV). The spontaneous emulsification method was used to generate the nanoemulsions in the study by Pavlović et al., and Tween 20 was utilized as the surfactant [36]. On the other hand, in our study, nanoemulsions were prepared by emulsification followed by high-speed homogenization, and a combination of Tween 80 and Span 80 was used as the surfactant system. Compared with the nanoemulsion reported by Pavlović et al., our nanoemulsion exhibited a smaller droplet size despite containing a higher RO-EO concentration. Although the PDI obtained in our study was higher than that of Pavlović et al., it remained below 0.3, indicating an acceptable and relatively narrow droplet size distribution for nanoemulsions. Furthermore, the higher absolute zeta potential of our nanoemulsion suggested improved colloidal stability compared with the nanoemulsion prepared by Pavlović et al. [36]. These findings indicate that differences in surfactant composition and preparation conditions may have contributed to the formation and stabilization of smaller droplets. Morphological investigation of nanoemulsions was conducted with TEM (Figure 3, Figure 4 and Figure 5). These findings revealed predominantly spherical droplets in formulations F1, F2, and F3, confirming the successful formation of nanoemulsions.

The antibacterial activities of TV-EO and RO-EO have been previously demonstrated in several studies [37,38,39]. The TV-EO exhibited inhibitory activity against *E. faecalis* and *C. albicans*, with MIC values of 3.125 mg/mL and 1.56 mg/mL, respectively. A previous study reported that a linalool chemotype of TV containing 76.15% linalool exhibited an MIC value of 1250 µg/mL against *C. albicans* [12]. Considering that the TV-EO used in the present study contained 79.51% linalool, the observed antifungal activity appears comparable to that reported for other linalool-rich thyme chemotypes. However, antimicrobial potency may vary considerably even among linalool-rich EOs. Hajlaoui et al. reported substantially lower MIC values against *E. faecalis* and *C. albicans* for *Coriandrum sativum* EO containing 76.41% linalool [40]. These differences suggest that antimicrobial activity cannot be explained solely by the abundance of the major constituent but may also be influenced by minor constituents and their synergistic, additive, or antagonistic interactions [41]. In our study, the antimicrobial activity results further highlighted the importance of EO composition. When tested in their free form, RO-EO exhibited lower MIC values against the tested bacterial strains than TV-EO, indicating stronger antibacterial activity. In the present study, RO-EO exhibited MIC values of 1.56, 0.78, and 1.56 mg/mL against *S. mutans*, *L. acidophilus*, and *E. faecalis*, respectively. GC-MS-FID analysis revealed that eucalyptol (58.13%) and camphor (10.80%) were the major constituents of this oil. These findings are generally consistent with previous reports describing the antimicrobial activity of eucalyptol-rich rosemary EOs. Kabotso et al. reported an MIC value of 6.25 mg/mL against *S. mutans* using an RO-EO containing 42.68% eucalyptol [42], whereas Mohamed Abdoul-Latif et al. demonstrated marked activity of an RO-EO characterized by eucalyptol and camphor as the predominant constituents against *E. faecalis* [43]. The lower MIC values obtained in the present study may be associated with the higher proportion of eucalyptol in our RO-EO. Nevertheless, variations in chemotype, geographical origin, harvesting season, extraction method, and interactions among EO constituents may also contribute to differences in antimicrobial activity. A similar result was observed for the nanoemulsion formulations, where F2 demonstrated better antibacterial activity than F1 against *S. mutans*, *L. acidophilus*, and *E. faecalis*. These findings suggest that the antibacterial activity of the nanoemulsions was influenced not only by nanoscale formulation characteristics but also by the intrinsic antimicrobial activity of the incorporated EOs. In contrast, F3 exhibited lower antibacterial activity than F2 despite possessing the smallest droplet size. This observation may be explained by the lower concentration of each individual EO in F3, where both TV-EO and RO-EO were present at half the concentration used in the single-oil formulations. Therefore, droplet size alone does not appear sufficient to explain the antibacterial performance of the investigated nanoemulsions.

Previous studies have reported antifungal activities of linalool-, eucalyptol-, and camphor-rich EOs against *Candida* species through mechanisms involving disruption of cell membrane integrity and increased membrane permeability [12,40]. In this study, the antifungal activity results differed from the antibacterial activity profile observed against the tested bacterial strains. Both F1 and F2 exhibited the same antifungal activity against *C. albicans* (MIC: 1/64), whereas F3 showed lower activity (MIC: 1/16). Similar to the antibacterial findings, the superior physicochemical characteristics of F3, particularly its smaller droplet size, did not lead to an increased antimicrobial activity. This observation further supports the notion that droplet size alone is insufficient to explain the antimicrobial activity of the investigated nanoemulsions.

EOs may exert antibiofilm activity through multiple mechanisms, including inhibition of exopolysaccharide production, disruption of microbial adhesion, interference with quorum-sensing systems, and alteration of metabolic activity within biofilms. It has been shown that terpenoid compounds such as linalool, eucalyptol, and camphor possess antibiofilm properties against various microorganisms [44,45,46]. Although RO-EO and F2 exhibited stronger antibacterial activity against planktonic oral pathogens, F3 demonstrated the highest antibiofilm activity, reaching an inhibition level comparable to CHX. This finding suggests that antibacterial activity against planktonic cells and antibiofilm efficacy against mature biofilms may be influenced by different factors.

The superior antibiofilm activity of F3 may be associated with its markedly smaller droplet size, which could facilitate diffusion through the extracellular polymeric substance (EPS) matrix of mature biofilms and increase interactions between bioactive compounds and biofilm-embedded cells. In addition, nanoemulsification may improve the dispersion and delivery of hydrophobic EO constituents within the biofilm structure. Although F3 exhibited lower antibacterial activity than F2, its smaller droplet size together with the combined presence of TV-EO and RO-EO constituents may have contributed to its enhanced antibiofilm performance. Therefore, while EO composition appears to be a major determinant of antibacterial activity, both nanoemulsion characteristics and EO composition may contribute to antibiofilm efficacy [47].

In addition to droplet size, zeta potential may also influence the interaction of nanoemulsions with biofilm matrices. In our study, F3 exhibited a less negative zeta potential (−21.2 ± 1.6 mV) compared to F1 (−46.8 ± 2.2 mV) and F2 (−34.5 ± 5.2 mV). This situation may reduce the electrostatic repulsive force between the nanoemulsion droplets and the negatively charged biofilm matrix, but it may also indicate lower colloidal stability. The enhanced antibiofilm activity cannot be attributed solely to zeta potential, as no direct evidence from biofilm penetration studies is available. The enhanced antibiofilm activity of F3 may be attributed to its smaller droplet size and the combined composition of TV-EO and RO-EO. The combination of these chemically distinct EOs may have provided complementary antimicrobial effects, while the smaller droplet size may have facilitated closer interaction of the nanoemulsion droplets with the biofilm matrix.

Mature biofilms are protected by a negatively charged EPS matrix that acts as a tight diffusion barrier against hydrophobic compounds. Pure EOs are inherently lipophilic and highly insoluble in water; when physically mixed without surfactant stabilization, the free compounds (linalool, eucalyptol, and camphor) undergo macrophase separation. Furthermore, when these free droplets reach the EPS barrier, they may create a competitive blocking effect at the matrix pore entrances, thereby inhibiting each other’s diffusion. This mechanism may explain the lower antibiofilm activity observed in single-component formulations. Nanoemulsion systems can improve the aqueous dispersion of hydrophobic compounds and may facilitate their interaction with biofilm-embedded cells. Therefore, the enhanced antibiofilm activity observed for F3 may be associated with improved delivery of EO constituents within the biofilm matrix as well as its smaller droplet size. However, the exact mechanisms underlying the superior antibiofilm performance of F3 remain to be elucidated and warrant further investigation [48,49].

Nevertheless, the complaint of a change in taste is the most prevalent side effect of EO mouthwashes. There have also been cases involving temporary local reactions such as palatal erythema and rare allergic reactions to EOs [50]. Because of the risk of swallowing, mouthwashes are not recommended for children under the age of six [51].

The present study has some limitations. Although the nanoemulsions were comprehensively characterized in terms of droplet size, PDI, zeta potential, morphology, and antimicrobial and antibiofilm activities, their long-term stability, cytotoxicity, and in vivo efficacy were not investigated. Future studies should focus on evaluating these parameters, as well as incorporating the nanoemulsions into a mouthwash formulation.

## 4. Materials and Methods

### 4.1. GC-MS-FID Analysis of EOs

EOs of TV and RO (Florame^®^, Saint-Rémy-de-Provence, France) were used in this study. The TV and RO EOs were analyzed using comprehensive GC-MS-FID to determine their phytochemical composition. Before injection, the commercial EOs were diluted with hexane. An HP-5MS column (30 m × 0.25 mm) was used for GC-MS-FID, and helium was used as the carrier gas at a constant flow rate of 0.9 mL/min. The oven temperature was first set at 60 °C for one minute, then increased at a rate of three degrees Celsius per minute to 246 °C, and it was maintained for thirty minutes. The samples were injected in split mode (25:1) at a volume of 1 μL. At the end of the separation, analytes were simultaneously directed to the MS and FID detectors using a splitter. Compound identification was based on spectral matching using the NIST17 mass spectral library and comparison of calculated retention indices (relative to an n-alkane series) with data from the literature and direct comparison with reference standards, when applicable. Peak area integration from the FID chromatograms was used to calculate the relative percentages of the identified compounds; reference substances were utilized when applicable [52].

### 4.2. Preparation of Nanoemulsions

TV-EO and RO-EO nanoemulsions were prepared by an emulsification method followed by high-speed homogenization using a homogenizer (IKA T10 Basic Ultra-Turrax, Staufen, Germany). The aqueous phase consisted of 2% (*w*/*v*) Tween 80 (Sigma-Aldrich, Steinheim, Germany) and distilled water, whereas the oil phase consisted of 5% (*w*/*v*) EO and 2% (*w*/*v*) Span 80 (Sigma-Aldrich, Germany). The compositions of the nanoemulsions are indicated in Table 7. F1, F2, and F3 contained 5% TV-EO, 5% RO-EO, and a combination of 2.5% TV-EO and 2.5% RO-EO, respectively. Each nanoemulsion was prepared using 25 mL of distilled water. F1, F2, and F3 formulations were prepared using 1.25 g TV-EO, 1.25 g RO-EO, and 0.625 g TV-EO together with 0.625 g RO-EO, respectively.

To obtain nanoemulsions, the aqueous and oil phases were mixed separately using a magnetic stirrer at 600 rpm for 5 min. Subsequently, the oil phase was added dropwise to the aqueous phase under continuous homogenization (speed setting 4) in an ice bath, and homogenization was continued for 10 min [53].

### 4.3. Characterization of Nanoemulsions

#### 4.3.1. Droplet Size and Distribution

The dynamic light scattering (DLS) technique was applied to measure the mean droplet size and PDI of nanoemulsions using the Litesizer 500 particle analyzer (Anton Paar, Graz, Austria). All formulations were diluted ten times with distilled water before measurement. The measurements were carried out with a disposable cuvette at a 90° angle and at room temperature (25 °C). The results are given as mean ± SD [54].

#### 4.3.2. Zeta Potential

A Litesizer 500 (Anton Paar, Graz, Austria) was utilized to determine the zeta potential of nanoemulsions using the Laser Doppler Velocimetry method. Prior to measurement, all formulations were diluted ten times with distilled water. The measurements were performed with an Omega cuvette using Kalliope software version 2.8.3., and the findings are presented as mean ± SD [55].

#### 4.3.3. Morphological Investigation

TEM was used for morphological investigation. TEM imaging was conducted utilizing a Talos L120C (Thermo-Fisher Scientific, Waltham, MA, USA). The formulations were set on carbon-coated TEM copper grids and allowed to air dry prior to analysis. The grids were inserted into the TEM after drying, and images were obtained at various magnifications [54].

#### 4.3.4. Antimicrobial Activity Tests

The antimicrobial activity of the TV-EO, RO-EO, and nanoemulsion formulations was assessed using the broth microdilution method to determine the MIC values [56,57]. The test organisms included *Streptococcus mutans* ATCC 25175, *Lactobacillus acidophilus* ATCC 4356, and *Enterococcus faecalis* ATCC 29212 as bacterial strains, and *Candida albicans* ATCC 10231 as the fungal strain. The EOs were dissolved in 5% DMSO.

For the antibacterial activity test, serial two-fold dilutions of the nanoemulsion formulations and EO samples (ranging from 50 to 0.39 mg/mL) were prepared in Mueller-Hinton Broth (MHB) (Merck, Darmstadt, Germany). Inocula were prepared from 24 h subcultures and adjusted to a 0.5 McFarland standard (approximately 1 × 10^8^ CFU/mL). The bacterial suspensions were then diluted in the MHB to obtain a final inoculum concentration of 5 × 10^5^ CFU/mL. After incubation at 35 °C for 18–24 h, the MIC value (mg/mL) was determined as the lowest concentration that completely inhibited visible bacterial growth. Negative controls consisted of 5% DMSO, MHB, and blank nanoemulsion formulation, while CHX (0.2%) was used as a positive control.

For the antifungal activity test, serial two-fold dilutions of the nanoemulsion formulations and EO samples (ranging from 50 to 0.39 mg/mL) were prepared in RPMI 1640 broth (ICN-Flow, Aurora, OH, USA). Fungal inocula were prepared from 24 h subcultures and adjusted to a 0.5 McFarland standard (approximately 1–5 × 10^6^ CFU/mL). The suspensions were then diluted in the RPMI 1640 broth to obtain a final inoculum concentration of 0.5 to 2.5 × 10^3^ CFU/mL. The microplate was incubated at 35 °C for 48 h. The MIC value (mg/mL) was defined as the lowest concentration that completely inhibited visible fungal growth. Negative controls consisted of 5% DMSO, RPMI 1640 broth, and blank nanoemulsion formulation, while CHX (0.2%) was used as the standard antifungal agent.

#### 4.3.5. Antibiofilm Activity Tests

The antibiofilm activity of the TV-EO, RO-EO, and nanoemulsion formulations was evaluated using the crystal violet assay, based on an in vitro microplate-based biofilm model. Before the test, the MIC values of the nanoemulsion formulations and EOs were determined against *Streptococcus mutans* ATCC 25175 [56,57]. For biofilm formation, *S. mutans* was cultured in Brain Heart Infusion (BHI) broth supplemented with 2% (*w*/*v*) sucrose. Final inoculum suspensions (∼10^6^ CFU/mL) were prepared in the respective media. In total, 100 µL of the inoculum suspension was added to 96-well microtiter plates for all test conditions. The plates were incubated at 35 °C for 72 h to allow the formation of mature biofilms. After biofilm formation, the medium was aspirated, and non-adherent cells were removed by washing the wells with sterile phosphate-buffered saline (PBS, pH 7.2). Subsequently, 100 µL of TV-EO, RO-EO, and nanoemulsion formulations were added to each well containing the mature biofilms. The plates were incubated at 35 °C for 24 h. After incubation, the wells were washed with PBS to remove non-adherent cells and dried at room temperature for 1 h. An amount of 100 µL of 0.5% crystal violet solution was added to each well to stain the biofilm cells. After 30 min, the wells were washed three times with PBS, and a 30:70 (*v*/*v*) acetone–alcohol solution was added to dissolve the bound dye within the biofilm matrix. BHI broth enriched with 2% sucrose was used as the control. The optical density (OD) of the dissolved crystal violet was measured at 620 nm using a microplate reader (CLARIOstar, BMG LABTECH GmbH, Ortenberg, Germany). The percentage biofilm inhibition values were calculated using the following formula:

Biofilm Inhibition (%) = [(OD (Growth control) − OD (Sample))/OD (Growth control)] × 100

Data are represented as means ± SD. Statistical differences were analyzed using Oneway ANOVA and Tukey’s test for post-hoc comparisons with GraphPad Prism software (version 8.0, GraphPad, Boston, MA, USA).

## 5. Conclusions

In conclusion, the present study demonstrated that nanoemulsions containing TV-EO and RO-EO can be successfully formulated and exhibit promising antimicrobial and antibiofilm activities against oral pathogens. The results showed that EO composition significantly influenced both the physicochemical characteristics and biological performance of the formulations. While F2 exhibited the strongest antibacterial activity, F3 demonstrated the highest antibiofilm efficacy, reaching an inhibition level comparable to CHX. These findings indicate that antibacterial and antibiofilm activities may be governed by different formulation-related factors and that droplet size alone is insufficient to explain biological activity. Overall, TV-EO and RO-EO nanoemulsions appear to be promising candidates for the development of natural mouthwash products targeting oral biofilms. However, their safety and tolerability have not yet been established in clinical settings. Therefore, further studies evaluating long-term stability, cytotoxicity, in vivo efficacy, patient acceptability (e.g., taste and mouthfeel), oral mucosal irritation, and overall tolerability are warranted to confirm their suitability for clinical application.

## Figures and Tables

**Table 1:** Phytochemical analysis results of TV-EO with GC-MS-FID.

	R.T	RRI Exp.	RRI Lit.	RRI Ref.	Compounds	%	IM
1	8.65	933	933	933	α-Pinene	1.46 ± 0.11	MS, RI, RS
2	9.16	947	948	947	Camphene	0.22 ± 0.02	MS, RI, RS
3	10.59	988	991	988	β-Myrcene	1.48 ± 0.10	MS, RI, RS
4	11.98	1011	1011		δ-3-carene	1.33 ± 0.09	MS, RI,
**5**	**13.39**	**1053**	**1059**		**γ-Terpinene**	**2.90 ± 0.20**	**MS, RI,**
6	13.79	1065	1066		Sabinene hydrate	0.51 ± 0.03	MS, RI,
7	13.95	1070	1075		Linalool oxide	0.43 ± 0.04	MS, RI,
8	14.59	1089	1088	1091	Terpinolene	0.62 ± 0.15	MS, RI, RS
**9**	**15.91**	**1108**	**1104**	**1105**	**Linalool**	**79.51 ± 0.98**	**MS, RI, RS**
10	18.21	1175	1171	1174	*endo*-Borneol	0.56 ± 0.02	MS, RI, RS
**11**	**18.76**	**1188**	**1182**	**1184**	**Terpinene-4-ol**	**3.64 ± 0.03**	**MS, RI, RS**
12	19.26	1199	1201	1198	α-Terpineol	0.48 ± 0.02	MS, RI, RS
**13**	**21.95**	**1262**	**1259**	**1259**	**Linalyl acetate**	**3.09 ± 0.02**	**MS, RI, RS**
14	23.81	1305	1305	1304	Thymol	0.63 ± 0.03	MS, RI, RS
15	28.93	1428	1426	1428	β-Caryophyllene	0.95 ± 0.03	MS, RI, RS
					**Total identified**	97.81%	

RT: Retention time. RRI exp: Linear program retention indices determined in this study. RRI lit: retention indices from the literature. RRI Ref: retention indices from reference substances. IM: Identification method. MS: Mass Library. RI: Comparison of RI values with the literature. RS: Reference substance.

**Table 2:** Phytochemical analysis results of RO-EO with GC-MS-FID.

	R.T	RRI Exp.	RRI Lit.	RRI Ref.	Compounds	%	IM
**1**	**8.74**	**935**	**933**	**933**	**α-Pinene**	**10.09 ± 0.02**	**RI, MS, RS**
**2**	9.21	949	948	947	Camphene	3.35 ± 0.00	RI, MS, RS
**3**	10.25	978	978		β-Pinene	6.93 ± 0.00	RI, MS
**4**	10.61	989	991	988	β-Myrcene	1.16 ± 0.01	RI, MS, RS
**5**	**12.63**	**1030**	**1032**	**1031**	**Eucalyptol**	**58.13 ± 0.04**	**RI, MS, RS**
**6**	13.42	1054	1058		γ-Terpinene	1.84 ± 0.01	RI, MS
**7**	15.17	1105	1104	1105	Linalool	0.53 ± 0.00	RI, MS, RS
**8**	**17.32**	**1155**	**1150**	**1150**	**Camphor**	**10.80 ± 0.03**	**RI, MS, RS**
**9**	18.16	1174	1176	1174	*endo*-Borneol	1.68 ± 0.02	RI, MS, RS
**10**	18.57	1184	1186	1184	Terpinene-4-ol	0.46 ± 0.00	RI, MS, RS
**11**	19.22	1199	1201	1198	α-Terpineol	1.00 ± 0.01	RI, MS, RS
**12**	23.21	1290	1288	1291	Bornyl acetate	0.40 ± 0.00	RI, MS, RS
**13**	27.04	1381	1377		α-Copaene	0.12 ± 0.00	RI, MS
**14**	28.95	1425	1425	1428	β-Caryophyllene	2.01 ± 0.00	RI, MS, RS
**15**	30.28	1460	1458		Humulene	0.22 ± 0.00	RI, MS
					**Total identified**	98.72%	

RT: Retention time. RRI exp: Linear program retention indices determined in this study. RRI lit: retention indices from the literature. RRI Ref: retention indices from reference substances. IM: Identification method. MS: Mass Library. RI: Comparison of RI values with the literature. RS: Reference substance.

**Table 3:** Droplet size, PDI, and zeta potential of nanoemulsions (mean ± SD, n = 3).

Formulation Code	Droplet Size (nm)	PDI	Zeta Potential (mV)
F1	161.18 ± 3.24	0.252 ± 0.020	−46.8 ± 2.2
F2	111.03 ± 0.69	0.215 ± 0.013	−34.5 ± 5.2
F3	55.40 ± 0.44	0.284 ± 0.009	−21.2 ± 1.6

**Table 4:** MIC values of the TV-EO and RO-EO (mg/mL).

Essential Oil	Bacteria			Fungus
	*S. mutans* ATCC 25175	*L. acidophilus* ATCC 4356	*E. faecalis* ATCC 29212	*C. albicans* ATCC 10231
TV-EO	3.125	1.56	3.125	1.56
RO-EO	1.56	0.78	1.56	3.125
TV + RO-EOs	0.78	0.78	1.56	3.125

**TV-EO:** *Thymus vulgaris* Essential Oil, **RO-EO:** *Rosmarinus officinalis* Essential Oil, **ATCC:** American Type Culture Collection.

**Table 5:** MIC values of the nanoemulsion formulations (1/4 to 1/512).

Formulation Code	Bacteria			Fungus
	*S. mutans* ATCC 25175	*L. acidophilus* ATCC 4356	*E. faecalis* ATCC 29212	*C. albicans* ATCC 10231
F1	1/128	1/128	1/64	1/64
F2	1/256	1/256	1/128	1/64
F3	1/16	1/32	1/32	1/16
F4	-	-	-	-
CHX (0.2%)	˂1/512	˂1/512	˂1/512	1/64

**F4:** Blank formulation (without EO), **CHX:** Chlorhexidine, **ATCC:** American Type Culture Collection, ‘-’**:** represents no activity. The values represent the dilution factors of the nanoemulsion formulations tested. The composition of each formulation is provided in Section 4.2.

**Table 6:** Biofilm inhibition (%) values of TV-EO, RO-EO, and nanoemulsion formulations. Data are presented as mean ± SD from four independent experiments (n = 4).

Sample	Biofilm Inhibition (%) (Mean ± SD)
TV-EO	67.11 ± 5.92
RO-EO	59.71 ± 10.51
TV + RO-EOs	47.96 ± 19.04
F1	65.11 ± 16.68
F2	60.35 ± 11.95
F3	80.55 ± 2.33
CHX	79.95 ± 1.49

**TV-EO:** *Thymus vulgaris* Essential Oil, **RO-EO:** *Rosmarinus officinalis* Essential Oil, **CHX:** Chlorhexidine.

**Table 7:** Composition of nanoemulsions.

Formulation	TV-EO * (*w*/*v*%)	RO-EO ** (*w*/*v*%)	Span 80 (*w*/*v*%)	Tween 80 (*w*/*v*%)	Distilled Water (mL)
**F1**	5	-	2	2	25
**F2**	-	5	2	2	25
**F3**	2.5	2.5	2	2	25

* TV-EO: *Thymus vulgaris* Essential Oil, ** **RO-EO:** *Rosmarinus officinalis* Essential Oil.

## Data Availability

Data is contained within the article.

## Abbreviations

The following abbreviations are used in this manuscript:

EO	Essential oil
TV	*Thymus vulgaris*
RO	*Rosmarinus officinalis*
PDI	Polydispersity index
TEM	Transmission electron microscopy
CHX	Chlorhexidine
DLS	Dynamic light scattering
SD	Standard deviation
DMSO	Dimethyl sulfoxide
BHI	Brain heart infusion
PBS	Phosphate-buffered saline
OD	Optical density
MIC	Minimum inhibitory concentration
ATCC	American Type Culture Collection
EPS	Extracellular polymeric substance
